# Developing a conceptual framework for the health protection of United Nations peacekeepers against the COVID-19 pandemic from global health perspectives

**DOI:** 10.1186/s41256-022-00280-0

**Published:** 2022-11-28

**Authors:** Quan Yuan, Yong Chen, Jiqing Wan, Rui Zhang, Miaomiao Liao, Zhaogang Li, Jiani Zhou, Ying Li

**Affiliations:** 1 Department of Social Medicine and Health Service Management, Army Medical University (Third Military Medical University), Chongqing, China; 2 National Engineering Research Center of Immunological Products, Department of Microbiology and Biochemical Pharmacy, College of Pharmacy, Army Medical University, Chongqing, China

**Keywords:** COVID-19, UN peacekeepers, Global health, Conceptual framework

## Abstract

The coronavirus disease 2019 (COVID-19) pandemic has posed particular health risks to United Nations peacekeepers, which require prompt responses and global attention. Since the health protection of United Nations peacekeepers against the COVID-19 pandemic is a typical global health problem, strategies from global health perspectives may help address it. From global health perspectives, and referring to the successful health protection of the Chinese Anti-Ebola medical team in Liberia, a conceptual framework was developed for the health protection of United Nations peacekeepers against the COVID-19 pandemic. Within this framework, the features include multiple cross-borders (cross-border risk factors, impact, and actions); multiple risk factors (Social Determinants of Health), multiple disciplines (public health, medicine, politics, diplomacy, and others), and extensive interdepartmental cooperation. These strategies include multiple phases (before-deployment, during-deployment, and post-deployment), multi-level cooperation networks (the United Nations, host countries, troop-contributing countries, the United Nations peacekeeping team, and United Nations peacekeepers), and concerted efforts from various dimensions (medical, psychological, and social).

## Background

Peacekeeping by the United Nations (UN) helps countries attrited by wars to create conditions for lasting peace, which has been proven to be one of the most effective ways to help countries navigate the difficult path from conflict to peace [1]. UN peacekeepers often face life-threatening health risks such as incidents, infectious or other diseases, and psychological trauma [2–5]. From 2000 to 2017, 2042 UN peacekeepers died, of which 879 deaths were caused by infectious or other diseases (43%), far more than the number of deaths caused by incidents (602, 29.5%) and violence (407, 19.9%) [6]. Therefore, health protection for UN peacekeepers is of significant importance. Since January 2020, the World Health Organization (WHO) has identified the coronavirus disease 2019 (COVID-19) pandemic as a public health emergency of international concern [7]. The outbreak of COVID-19 poses a new threat and has multiple impacts on UN peacekeepers’ health protection [8]. For example, by October 1, 2020, there were 463 COVID-19 cases, including three deaths among Minusca peacekeepers in the Central African Republic, and by November 25, 2020, there were 171 COVID-19 cases, including six fatalities among Monusco peacekeepers in the Democratic Republic of Congo [9]. To deal with the challenges of the COVID-19 pandemic faced by UN peacekeepers, comprehensive measures should be taken to prevent infection and protect their health promptly.

There are several measures for protecting the health of UN peacekeepers against COVID-19. At the beginning of the outbreak, the UN Security Council resolved in March 2020 to address the security issues faced by UN peacekeepers and emphasize their health and safety protection [10]. Given that UN peacekeepers’ cross-border movement might bring COVID-19 transmission risks to host countries [11], the UN took measures, including restricting UN peacekeepers’ cross-border rotation, requiring them to maintain social distance, and requesting their constant attention on the latest epidemic trends [12]. The UN came up with the *Core Pre-deployment Training Materials*, the *Course Handbook*, and the *Student Handbook for instructors on COVID-19 pre-deployment awareness training* in October 2020, providing essential knowledge for UN peacekeepers on epidemic protection, such as personal hygiene measures, quarantine, and trainees’ responsibilities for self-protection [13, 14]. Furthermore, the UN coordinated COVID-19 humanitarian vaccination for peacekeepers and citizens in host countries, as the coronavirus kept mutating into Delta, Omicron, and other variants [15, 16]. However, host countries, the UN, and UN peacekeepers have limited knowledge and capacity to fight the COVID-19 pandemic [17]: host countries seem to have inefficient medical and health systems for UN peacekeepers’ health protection against COVID-19 [18]. Although there are training materials provided for peacekeepers by the UN, knowledge, and training regarding features of COVID-19 seem insufficient; UN peacekeepers from different countries with various cultural backgrounds may have conflicting perceptions and take inconsistent actions against the COVID-19 pandemic [19].

The health protection of UN peacekeepers against the COVID-19 pandemic and other infectious diseases such as Ebola and Avian Influenza are considered a global health problem [20, 21]. Strategies from global health perspectives may help address these problems. For example, no members of the Chinese Anti-Ebola medical team deployed to provide vital medical care in Liberia were infected because comprehensive and multi-disciplinary measures were taken from global health perspectives [3]. Similarly, comprehensive measures taken from global health perspectives may support the health protection of UN peacekeepers against the COVID-19 pandemic [22, 23]. Therefore, a conceptual framework with features to understand the issue and strategies to address it may be necessary for UN peacekeepers’ health protection against COVID-19. The purpose of this study is to develop such a framework containing features and strategies for the health protection of UN peacekeepers against the COVID-19 pandemic, with the guidance of global health characteristics and global health governance [24–33] and the experiences of the Chinese medical team’s health protection during the Ebola pandemic in Liberia [3].

## Global health and global health governance

Global health is the study of health problems, health issues, and health concerns the impact of which may transcend national borders, and it may be affected by the environment and experience of other countries, to promote health for all [24–26]. Changes in global health governance require extensive multi-disciplinary cooperation within and outside the health fields and international collaborative actions crossing national borders [27]. Global health has the following characteristics: (1) *Cross-border risk factor*s, impact, and collective efforts. Since global health may be affected by the factors of other countries, the risk factors are cross-borders. Global health has a cross-border impact because it can be transferred to other countries. Different countries across borders are required to bring useful insights and take collective action to address global health problems [28, 29]. (2) *Interdisciplinary cooperation*. Professionals from various disciplines inside and outside health can cooperate and contribute to improving global health [30, 31]. *(3) Interdepartmental cooperation*. Wide social determinants of health (SDH) are associated with global health problems, and consequently, it is essential to utilize interdepartmental cooperation among health, political, cultural, educational, and other departments [32, 33].

Global health governance refers to using formal and informal institutions, rules, and processes to deal with global health problems [34]. (1) *Establishing cooperative networks to strengthen global health systems*. Action should be taken from bilateral, regional, and multilateral perspectives to facilitate the establishment of a cooperative network among governments, intergovernmental organizations (IGOs), nongovernmental organizations (NGOs), public-private partnerships (PPPs), and others [35]. To strengthen global health systems, it is vital to implement important treaties on global health problems, produce effective responses to global health threats, and prevent health problems from becoming global dangers [36]. (2) *Enabling multiple efforts from various perspectives.* By considering the social determinants of health, multiple efforts should be made to address global health issues from political, economic, and social contexts to the local, neighborhood, and household levels [37]. It also involves implementing international regulations and laws from security, economics, human rights, and other scopes to seek multipurpose surveillance and intervention against global health problems [38].

## Features of health protection among UN peacekeepers against the COVID-19 pandemic

### Multiple cross-border factors related to the COVID-19 infection and control among UN peacekeepers

(1) *Cross-borders* Global health problems have cross-border risk factors and require countries to take collective action to address them [28]. UN peacekeepers who travel across borders to complete peacekeeping missions face potential risks of contracting COVID-19 [39]. The impact of COVID-19 among UN peacekeepers is cross-border because the COVID-19 pandemic could impact peacekeepers’ completion of missions and threaten the health of contacts at home and abroad. Therefore, collective actions from troop-contributing countries, host countries, and the UN would be more effective in COVID-19 prevention and control among UN peacekeepers. (2) *Multiple factors* During the Ebola pandemic in Liberia, the Chinese medical team faced and identified multiple risk factors, including resource-limited living and working environment, the lack of social networks and the experience of political unrest, an underdeveloped local public health system, and anxiety and fear of contracting infectious diseases [3]. During the COVID-19 pandemic, they face multiple risk factors, including poor natural environments, such as extreme weather and natural disasters, and backward industrial environments, such as unsanitary water use and the lack of medical facilities [40]. UN peacekeepers’ diverse health education, health literacy, family backgrounds, and income levels can lead to different perceptions and actions regarding health protection against COVID-19 [41]. The health protection of UN peacekeepers against COVID-19 is influenced by their health conditions, including lifestyle and disease. Many UN peacekeepers have mental health problems like depression, anxiety, and infectious diseases like HIV and AIDS. It was reported that 20% of Australian veterans of UN peacekeepers in Somalia had problems with anger control, 29% of Canadian peacekeeping veterans had depressive disorders after deployment, and in Sub-Saharan Africa, mandatory HIV testing was reported by 71%, occurring before deployments and peacekeeping missions [42–45]. In addition, access to health facilities, the availability of vaccinations, the health condition of local vulnerable groups such as children, women, and the elderly, and the use of personal protective equipment (PPE) such as facial masks and other personal hygiene products can also be regarded as risk factors in UN peacekeepers’ health protection against COVID-19 [46, 47].

### Multiple interdisciplines in health protection against the COVID-19 pandemic among UN peacekeepers

Global health has interdisciplinary characteristics that require professionals from various disciplines to cooperate in solving global health problems [25]. Global health is dedicated to improving health equity for all human beings and integrating health protection into all policies by involving various disciplines in the policy-making process and initiating cooperation among various disciplines [30–32]. The health protection of the Chinese Anti-Ebola medical team in Liberia has a multi-disciplinary feature involving experts with public health credentials, professionals from clinical medicine, preventive medicine, biology, laws, management, sociology, and other backgrounds.

Based on interdisciplinary characteristics in global health and the multi-disciplinary feature in the health protection of the Chinese Anti-Ebola medical team in Liberia, the health protection of UN peacekeepers against COVID-19 has multiple interdisciplinary features. (1) *Public health.* Promoting health for all and interdepartmental cooperation regarding global health characteristics can help solve emergent public health crises. For example, the Chinese Anti-Ebola medical team in Liberia involved experts with public health credentials for health protection [3]. The health protection of UN peacekeepers requires insights from the public health discipline to prevent coronavirus infections in large groups [48]. (2) *Medicine.* With the aim of achieving health for all, referring to the experience of the Chinese Anti-Ebola medical team in Liberia [3], experts from clinical medicine, preventive medicine, and other medical backgrounds can help provide UN peacekeepers with medical treatment, including diagnosis, prescriptions, injections, transfusions, and surgeries during the COVID-19 pandemic [49, 50]. (3) *Politics.* Global health is dedicated to improving health equity for all human beings and integrating health protection into policies [30–32]. For instance, the Chinese medical team referred to successful policies and procedures for disease in their health protection in Liberia [3]. In response to the COVID-19 pandemic, political bodies could look at this problem cooperatively from a global standpoint and take political actions such as implementing regulations and laws to protect UN peacekeepers’ health [22]. (4) *Diplomacy.* Global health requires countries to take collective action to address global health issues across borders [28]. The Chinese medical team coordinated embassies and local Chinese enterprises to mitigate supply shortages during the Ebola pandemic in Liberia [3]. For the health protection of UN peacekeepers against the COVID-19 pandemic, it is critical to take humanitarian measures in health diplomacy from regional, bilateral, and multilateral perspectives in governments and organizations [51]. (5) *Others.* Based on the interdisciplinary features of global health characteristics, given experience in the health protection of Chinese medical teams in Liberia, disciplines such as biology, laws, management, and sociology could be used in UN peacekeepers’ health protection against the COVID-19 pandemic [3, 52].

### Multiple interdepartmental cooperation in health protection against the COVID-19 pandemic among UN peacekeepers

Global health facilitates interdepartmental cooperation between health, political, cultural, educational, and other departments. To address global health problems, it is essential to utilize interdepartmental collaboration regarding the social determinants of health and promote human health development [27, 29, 33]. During the Ebola pandemic in Liberia, the Chinese medical team not only utilized support from Chinese departments in government but also gained experience from local governments in Liberia and departments and organizations from other countries. By visiting the stadium in Monrovia and existing Ebola treatment centers from other countries in Liberia, the Chinese Anti-Ebola medical team addressed health problems from physical, social, and cultural perspectives [3].

Given the interdepartmental cooperation in global health and the relative experience of visiting local and foreign departments, the health protection of UN peacekeepers against the COVID-19 pandemic has multiple interdepartmental cooperation features, including cooperation among governments, intergovernmental organizations (IGOs), nongovernmental organizations (NGOs), and public-private partnerships (PPPs). (1) *Government*. Health departments in different countries could negotiate and develop strategies against the coronavirus, such as controlling viral transmission through trade and travel and implementing quarantine to reduce the burden on health systems [52, 53]. (2) *IGOs*. IGOs such as the H4 + partnership in the UN (WHO, World Bank, United Nations Population Fund, United Nations International Children’s Fund, and Joint United Nations Program on HIV/AIDS) could cooperate to ensure UN peacekeepers’ health during the COVID-19 pandemic [54]. The WHO established the Global Outbreak Alert and Response Network in response to significant and emergent infectious diseases [55]. (3) NGOs. NGOs that are not-for-profit and humanitarian in nature play a key role in reporting public health concerns and initiating global programs across borders for the health protection of UN peacekeepers against COVID-19. Charity group foundations such as the Bill & Melinda Gates Foundation, the International Committee of the Red Cross, and Doctors Without Borders, Medecins Sans Frontieres could support the health of UN peacekeepers during the COVID-19 pandemic [56]. (4) *PPPs*. PPPs, including the Global Alliance for Vaccines and Immunization (GAVI) and Think Tank, can cooperate in the health protection of UN peacekeepers, such as providing them with sufficient vaccines [57, 58]. (5) *Others*. Moreover, various human rights bodies, regional efforts, bilateral programs, and initiatives can contribute to UN peacekeepers’ health protection against COVID-19 [59]. For example, the COVAX project was established through the joint efforts of the GAVI Alliance, the Alliance for Epidemic Prevention Innovation, and the WHO to provide countries with vaccination support and improve medical availability among UN peacekeepers against the COVID-19 pandemic [60].

## Strategies for the health protection of UN peacekeepers against the COVID-19 pandemic

### Multi-phases of health protection against COVID-19 pandemic among UN peacekeepers

During the Ebola pandemic, the Chinese medical team took measures in three phases (before deployment, during deployment, and post deployment) to protect their health. Before deployment, the Chinese medical team had the experience of attending a 1.5-month intensive health training program on the procedures of Ebola [3]. During deployment, the Chinese medical team had experience implementing a three-level safety supervision program for the health protection of all staff [3]. After deployment, the Chinese Anti-Ebola medical team had the experience of being isolated for 21 days, being provided with a nutritional diet and psychological counseling, and receiving expert observation of signs and symptoms [3].

In response to the COVID-19 pandemic, measures should be taken in three phases (before deployment, during deployment, and post-deployment) to protect the health of UN peacekeepers. (1) *Before deployment.* Troop-contributing countries could improve their health protection knowledge and skills and take preventive measures such as physical examination, health education, psychological counseling, vaccination, and PPE preparation before deployment [61]. (2) *Duration of deployment*. It is necessary to establish effective global health systems that can provide sufficient medical and health services for UN peacekeepers, help them develop healthy life habits, and organize health lectures on the coronavirus during deployment [62]. (3) *Post-deployment*. To protect the health of UN peacekeepers after missions, it is necessary to pay attention to their health conditions and infection risks when deployed among larger populations. It is recommended to take preventive measures for transportation and quarantine when they arrive home [63].

### Multi-level cooperation networks of health protection measures against the COVID-19 pandemic among UN peacekeepers

Global health governance calls for establishing cooperative networks to strengthen global health systems from bilateral, regional, and multilateral perspectives among governments, organizations, and others [35]. To produce effective responses to global health problems, it is vital to implement important strategies within cooperative networks and global health systems [38]. The Chinese government and the local government in Liberia cooperated to ensure supplies for the Chinese Anti-Ebola medical team in Liberia [3]. During the Ebola pandemic, the National Ebola Command Center and many international organizations in Liberia were responsible for coordinating responses to the global health problem of Ebola [3].

Referring to the actions of establishing cooperative networks and strengthening global health systems in global health governance, and the experience of the health protection of the Chinese Anti-Ebola medical team in Liberia, it is suggested to use multi-level cooperation networks of health protection measures to strengthen global health systems against the COVID-19 pandemic among UN peacekeepers. Multilevel cooperation networks of health protection measures are required to enhance cooperation among the UN, host countries, troop-contributing countries, the UN peacekeeping team, and UN peacekeepers for health protection against the COVID-19 pandemic. (1) *The UN*. The UN could develop preventive strategies to strengthen the global health system against COVID-19 for the health protection of UN peacekeepers [64]. For instance, the Department of Operational Support, the Office of Military Affairs, and the Police Division in the UN can monitor the situation and advise governments according to the latest COVID-19 trends; the Office of Rule of Law and Security Institutions in the UN can prepare operational guidance to mitigate the transmission of the COVID-19; the UN Police can outline procedures for UN peacekeepers to follow in daily operations during the COVID-19 pandemic [65]. (2) *Host countries*. For the health protection of UN peacekeepers, host countries could improve public health systems, provide sufficient medical support, including medicines, and ensure the quality of medical services during COVID-19 [66, 67]. (3) *Troop-contributing countries*. Troop-contributing countries could prepare UN peacekeepers with health protection knowledge and skills before deployment, provide them with psychological and social support from home during deployment, and organize experts to observe signs and symptoms after deployment [43]. (4) *UN peacekeeping team*s. As a group, the UN peacekeeping team could train and evaluate the health protection capacity of peacekeepers and organize peer support for their health protection against the COVID-19 pandemic [68–70]. (5) *UN peacekeepers*. Individuals could be encouraged to take initiatives in their health protection against COVID-19 by learning related knowledge and skills, maintaining healthy lifestyles, etc. [43].

### Multiple efforts from various dimensions of health protection measures against the COVID-19 pandemic among UN peacekeepers

Based on global health governance, multiple efforts should be made to address global health issues in the political, economic, and social contexts at the local, neighborhood, and household levels [36]. Taking the social determinants of health into account, actions should involve various aspects, such as the implementation of international regulations and laws from security, economics, human rights, and other scopes, to seek multipurpose surveillance and intervention against global health problems [37]. During the Ebola pandemic in Liberia, the Chinese medical team used a bio-psycho-social model to protect their health from various aspects throughout their mission [3]. Given Liberia’s underdeveloped public health system, the Chinese Anti-Ebola medical team biologically paid attention to the disinfection of the environment and the use of PEE. Psychologically, they were provided with psychological counseling to ease their worries about diseases, and socially, they gained social support from countries, organizations, colleagues, friends, and families for their health protection and health diplomacy [3].

According to the actions of enabling multiple efforts from various aspects of global health governance and the experience of protecting the Chinese medical team during the Ebola pandemic in Liberia based on the bio-psycho-social model, multiple efforts from various dimensions, including medical, psychological, social, and other measures should be made to protect UN peacekeepers against COVID-19. (1) *From a medical perspective*, macroscopically, it is vital to improving the efficiency of local public health systems and the capacity of medical personnel for the health protection of UN peacekeepers during the COVID-19 pandemic. Organizing health training on COVID-19 diagnosis and treatment is recommended, emphasizing the importance of healthy eating and exercising habits and regulating the personal use of PPE, such as mask-wearing among UN peacekeepers [71, 72]. (2) *From the psychological dimension*, since fear, loneliness, anxiety, depression, post-traumatic stress, and other mental health symptoms are considered as causing psychological harm to UN peacekeepers during COVID-19 [73, 74], it is suggested to improve the peacekeepers’ access to psychological help, including face-to-face and online counseling services, and provide them with targeted psychological interventions against COVID-19, such as enhancing their awareness of symptoms, self-care, and peer-support [75, 76]. (3) *From the social dimension*, helping UN peacekeepers adapt to the local social and cultural environments could make them feel more familiar with and supported in completing missions during the COVID-19 pandemic [77]. Moreover, it is necessary to communicate with and learn from other countries based on health diplomacy, such as coordinating with embassies and visiting medical institutions from other countries [50]. This would assist the peacekeepers in having equipment inspection, logistics support, food supervision, and so on, to enhance their health protection during the COVID-19 pandemic [71].

## A generalized conceptual framework for recommendation

**Fig. 1:**
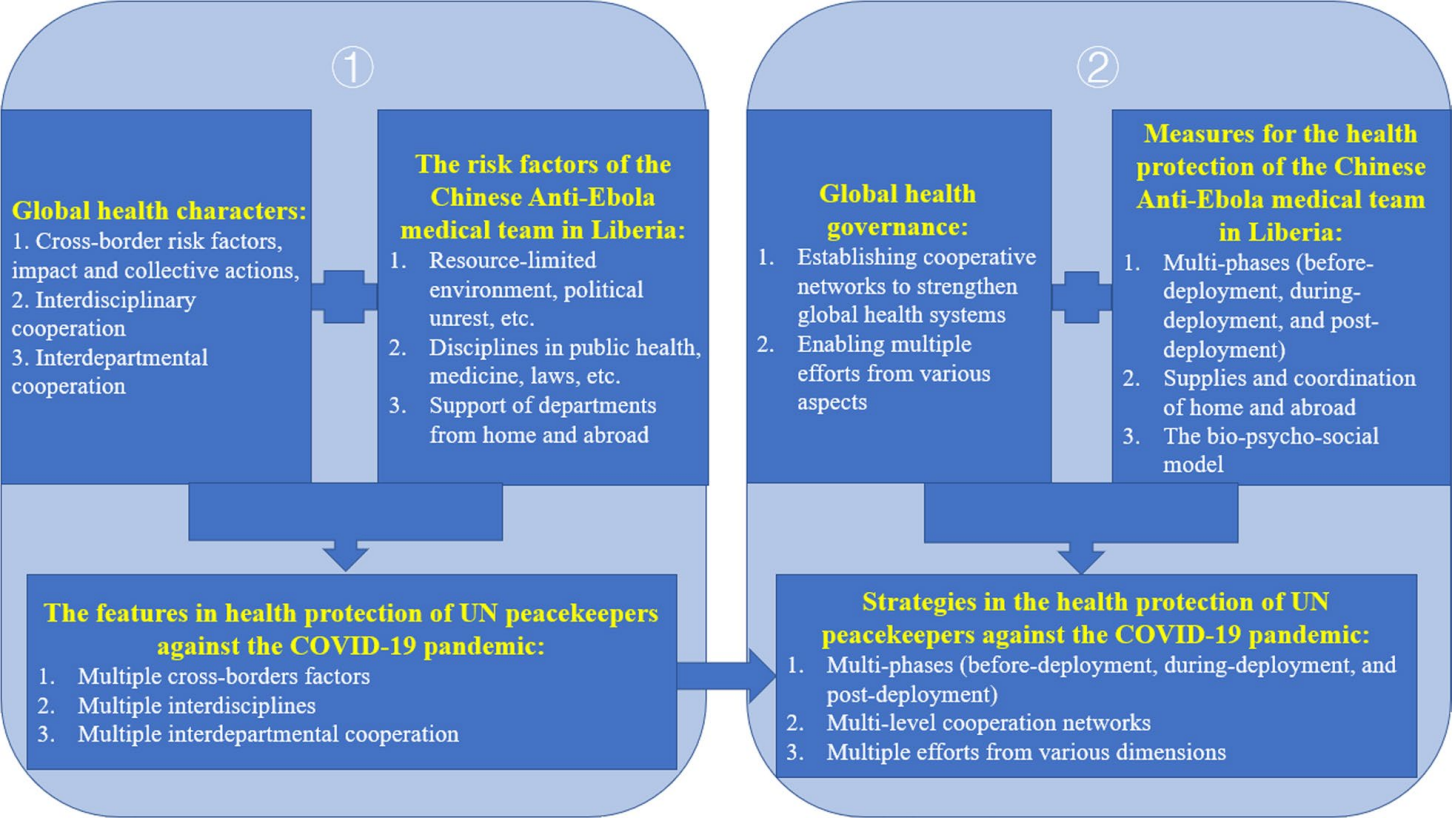
A conceptual framework of the health protection among UN peacekeepers against the COVID-19 pandemic from global health perspectives. This figure illustrates the development of a conceptual framework of health protection among UN peacekeepers against the COVID-19 pandemic from global health perspectives, and refers to the successful experience of the health protection of the Chinese Anti-Ebola medical team in Liberia. These features include multiple cross-borders, multiple interdisciplines, and multiple interdepartmental cooperation. These strategies include multi-phases, multilevel cooperation networks, and multiple efforts from various dimensions

The health protection of UN peacekeepers against the COVID-19 pandemic is a global health issue. From global health perspectives and referring to the successful experience of health protection of the Chinese Anti-Ebola medical team in Liberia, a conceptual framework can be developed for the health protection of UN peacekeepers against the COVID-19 pandemic (Fig. 1). The features include multiple cross-borders (cross-borders risk factors, impact, and actions, multiple risk factors such as SDH), multiple interdisciplines (public health, medicine, politics, diplomacy, and others), and multiple interdepartmental cooperation (IGOs, NGOs, PPPs, and others). These strategies include multiple phases (before-deployment, during-deployment, and post-deployment), multi-level cooperation networks (the UN, host countries, troop-contributing countries, the UN peacekeeping team, and UN peacekeepers), and multiple efforts from various dimensions (medical, psychological, and social).

## Data Availability

Not applicable.

## Abbreviations

COVID-19: Corona Virus Disease 2019
UN: United Nations
WHO: World Health Organization
IGOs: Intergovernmental Organizations
NGOs: Nongovernmental Organizations
PPPs: Public-private partnerships
PPE: Personal protective equipment
SDH: Social determinants of health
